# Revolutionizing Drug Delivery and Therapeutics: The Biomedical Applications of Conductive Polymers and Composites-Based Systems

**DOI:** 10.3390/pharmaceutics15041204

**Published:** 2023-04-10

**Authors:** Sharanya Paramshetti, Mohit Angolkar, Adel Al Fatease, Sultan M. Alshahrani, Umme Hani, Ankitha Garg, Gundawar Ravi, Riyaz Ali M. Osmani

**Affiliations:** 1 Department of Pharmaceutics, JSS College of Pharmacy, JSS Academy of Higher Education and Research (JSSAHER), Mysuru 570015, India; 2 Department of Pharmaceutics, College of Pharmacy, King Khalid University, Abha 61421, Saudi Arabia; 3 Clinical Pharmacy Department, College of Pharmacy, King Khalid University, Abha 61421, Saudi Arabia; 4 College of Applied Medical Sciences, Bisha University, Bisha 67714, Saudi Arabia; 5 Department of Pharmaceutical Quality Assurance, Manipal College of Pharmaceutical Sciences, Manipal Academy of Higher Education (MAHE), Manipal 576104, India

**Keywords:** conductive polymers, smart biomaterial, composites, electrochemical synthesis, drug delivery, biosensors, tissue engineering, neural probes

## Abstract

The first conductive polymers (CPs) were developed during the 1970s as a unique class of organic substances with properties that are electrically and optically comparable to those of inorganic semiconductors and metals while also exhibiting the desirable traits of conventional polymers. CPs have become a subject of intensive research due to their exceptional qualities, such as high mechanical and optical properties, tunable electrical characteristics, ease of synthesis and fabrication, and higher environmental stability than traditional inorganic materials. Although conducting polymers have several limitations in their pure state, coupling with other materials helps overcome these drawbacks. Owing to the fact that various types of tissues are responsive to stimuli and electrical fields has made these smart biomaterials attractive for a range of medical and biological applications. For various applications, including the delivery of drugs, biosensors, biomedical implants, and tissue engineering, electrical CPs and composites have attracted significant interest in both research and industry. These bimodalities can be programmed to respond to both internal and external stimuli. Additionally, these smart biomaterials have the ability to deliver drugs in various concentrations and at an extensive range. This review briefly discusses the commonly used CPs, composites, and their synthesis processes. Further highlights the importance of these materials in drug delivery along with their applicability in various delivery systems.

## 1. Introduction

Recently, a lot of effort has been put into developing novel drug-release systems that transport bioactive compounds from a reservoir to a host system while managing the pace and duration of delivery. The most effective way to administer the drug would be to deliver it to a specific area in the body where it is physiologically necessary. The system should also be able to recognize specific chemical species present in physiological media (such as pH or analyte concentration) and self-regulate their supply (a process known as chemical triggering), or it may be triggered externally, such as by light, near-infrared (NIR), or magnetically triggered drug delivery [1].

In recent decades, polymers, the most versatile type of materials, have revolutionized our lives. However, the differences between biomedical polymers that are transient or biodegradable, such as aliphatic polyesters, poly (ester amides), and polyphosphoesters, and those that are permanent or non-biodegradable, such as ultrahigh molecular weight polyethylene, polyethyleneimine, and poly (dimethyl siloxane), were identified 30 years ago. Due to their nonbiodegradability and bioinert characteristics, permanent polymers are employed as implantable prostheses for the partial or complete alternatives of the functions of damaged organs. Furthermore, a wide variety of transient polymers have also been investigated and characterized for various biomedical applications, such as controlled and sustained drug delivery systems, temporary platforms for cell culture and tissue regeneration, and absorbable wound healing devices [2]. Following that, pharmaceutical and polymer sciences developed unique drug delivery systems (DDSs) that were “novel” in terms of their physical state, shape, size, and flexibility. The main purpose of polymeric delivery systems is to control drug distribution either temporally or spatially. The first chemically synthesized polymer-based DDS (polyglycolic acid) was developed, which encouraged research into new biodegradable polymers. Thus, in order to boost the delivery system’s residence time and performance by directly interacting with an epithelial cell layer, bioadhesive polymers were developed.

Polymers used as biomaterials can be produced to have precise chemical, physical, interfacial, and biomimetic properties, allowing for a wide variety of applications. Polymers, as opposed to other types of biomaterials such as ceramics and metals, have the benefit of being able to be manufactured in a wide range of compositions with various structures and properties. Due to the structural diversity, selecting and designing a polymer is challenging and requires a thorough understanding of the polymer’s surface and bulk properties that can provide the necessary interfacial, mechanical, chemical, and biological functions. The selection of polymers is influenced by their physical characteristics as well as the necessity for extensive biochemical characterization and precise preclinical procedures to ensure their safety. This includes physical characteristics such as durability, permeability, and degradability, as well as surface characteristics like hydrophilicity, lubricity, surface energy, and smoothness, which influence biocompatibility with blood and tissues. The water sorption capacity of the polymers, which undergo hydrolytic breakdown and swelling, is also determined by their surface properties (hydrogels). On the contrary, water-repellent materials are required for long-term usages, such as dental and orthopedic implants, to keep the toughness and mechanical strength from deteriorating or eroding. Chemical, physical, and biological methods could be used to improve surface characteristics and boost biocompatibility. Enzymes, medicines, proteins, and antibodies have been grafted onto polymer surfaces to develop ‘polymer therapies’ that may be targeted to organs and cells. For controlled delivery systems, the bulk parameters to take into account include the adhesion, site of action, solubility, and molecular weight, depending on the release mechanism [3,4,5,6].

Implantable biomaterials in the future will be interactive and programmable, enabling effective communication with surrounding tissues. To govern cellular attachment, proliferation, and differentiation, materials that integrate stimulatory cues, such as electrical signals, can be used. For instance, it has been demonstrated that electrical fields can promote the healing of peripheral, spinal, and cranial nerves, skin and connective tissue, and bone. Hence, researchers have made an effort to incorporate electrical impulses directly into these biomaterials. Electroactive materials could be used to physically act as a template for cell growth and tissue regeneration while also providing an electrical stimulus to the injured area. For this intent, polymers have been modified to exhibit permanent charges (electrets) or produce transient surface charges (piezoelectric materials). In vitro and in vivo studies have shown that these compounds promote nerve and bone cell proliferation [7].

Electroactive biomaterials are a type of “smart biomaterial” that allows electrical, electromechanical, and electrochemical stimulation to be delivered directly to cells and/or tissues. Conductive polymers (CPs), photovoltaic materials, electrets, and piezoelectric are all examples of electroactive biomaterials. Without necessitating an external power supply, electrical stimulation (ES) can be supplied using electrets and piezoelectric materials, although control over the stimulus is restricted. Contrarily, CPs are capable of being synthesized to be porous, biocompatible, and biodegradable and offer good control of the electrical stimuli in addition to having both electrical and optical properties [7,8,9,10].

Researchers have been exploring intrinsically conductive polymers’ remarkable electrical characteristics for a multitude of applications since their discovery. These polymers have electrical features, such as low ionization potential, low energy optical transmission, and strong electron affinities, due to the conjugated-electron backbone. Light-emitting diodes, solar cells, electrochromic displays, supercapacitors, thin film transistors, and sensors can all benefit from these materials’ unique features. For these applications, conducting polymers has drawn the interest of numerous research groups. Researchers have recently become interested in conducting polymers and electroactive polymers to investigate their capability in biomedicine. This novel generation of “smart” biomaterials has been used to investigate as coatings of conventional electrode systems used in stimulation and neural sensing, biosensors, electrically induced DDS, modulators of bone, nerve, cardiac, and skeletal muscle cell activities, and emerging technologies, for instance, tissue engineering and regenerative medicine [11]. Polypyrrole (Ppy), polythiophene (PTh), and its derivatives, like poly (3, 4-ethylene dioxythiophene) (PEDOT) and polyaniline (PANI), are among the most studied conductive polymers in the biomedical field. The majority of studies have been done on in vitro testing and biocompatibility methodologies to better understand the polymer’s interaction with biological tissues. The fundamental problem in using conducting polymers for therapeutically relevant biomedical implants and devices has been tailoring them to have suitable electrical conductivity, processability, mechanical properties, and acceptable biocompatibility. The synthesis of composites of CPs incorporating conductive nanoparticles (NP) and non-conductive polymers to enhance biocompatibility and mechanical performance is one emerging technique to alleviate some of these limitations [12]. This review highlights the various CPs and their composite materials, their synthesis techniques, and their importance in drug delivery systems. Further, focuses on their application in the various biomedical field, such as drug delivery, tissue engineering, biosensors, bioactuators, neural probes, and wound healing.

## 2. Conductive Polymers

CPs are known to be organic materials with distinctive optical and electrical characteristics similar to those of inorganic metals and semiconductors. They are synthesized via simple, cost-effective, and flexible approaches. The electro-polymerization technique makes it simple to combine CPs into supramolecular structures with a variety of functions. To adapt and fine-tune the CPs for integration and interface into biomedical applications, such as biomaterials and biosensors, a wide range of methodologies have been established. Such discoveries are sought after by researchers in various fields of biomedicine, including bioengineering, biosensors, and regenerative medicine, as they may serve as the basis for future developments. CPs have shown promise in inducing a variety of cellular pathways, enhancing their potential in biomedical applicability. They have also sparked interest in various fields of biomedical because of their rapid responses to electric fields in diverse tissues. Conductive polymers have been employed to increase the speed, stability, and electrical sensitivity of a wide variety of biomedical devices and their interactions with biological tissues. One might anticipate that CPs could be qualified as suitable candidates for usage in a variety of biological and medical applications since CPs come in a wide variety of forms, such as conductive polymeric films, nanotubes, nanofibers, nanowires, scaffolds, neural probes, etc., and these have been proven to interact with biological substances while maintaining their biocompatibility [7,13,14,15,16].

**Figure 1:**
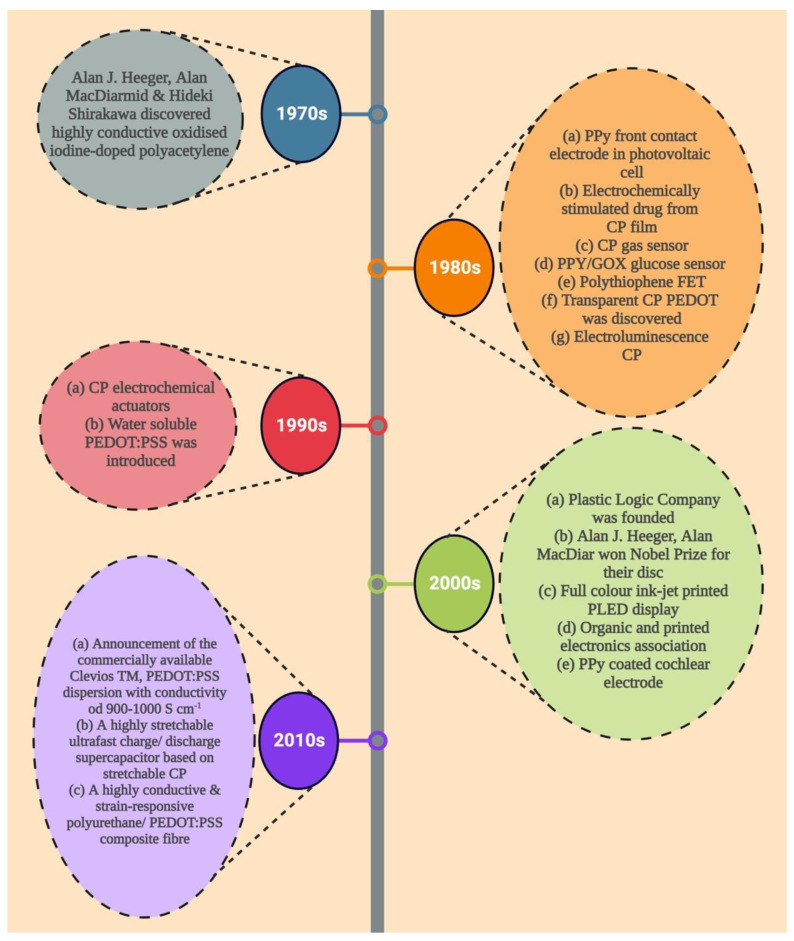
A brief overview of the history of conductive polymers and their application.

The discovery of poly (sulphur nitride) [(SN)x] in 1975, which superconducts at low temperatures, sparked interest in conductive polymer research [17]. Even though CP complexes, such as tetraoxalato-platinates, tetracyano, and Krogman salts charge transfer complexes, have previously been identified, MacDiarmid and Heeger’s rediscovery of polyacetylene in 1977 is significant; it was first discovered by Shirakawa and group in 1977 using a Ziegler Natta type of polymerization catalyst. By simply doping polyacetylene (10^−9^ S cm^−1^) with oxidizing agents (p-doping), such as I_2_, NOPF_6_, and AsF_5_, or reducing agents, such as sodium napthalide (n-doping), they were able to increase its electrical conductivity by many orders of magnitude to 10^5^ S cm^−1^ [18,19]. The electrodeposition of highly conductive, processable, and stable films of polypyrrole by Diaz et al. is a noteworthy accomplishment in this field. Numerous conducting polymers, such as polyacetylene, polyaniline, polypyrrole, polythiophene, poly (phenylene vinylene), polycarbazole, poly(3,4-ethylenedioxythiophene), polyphenylene, and polyfluorene, have been developed as a result of these ground-breaking discoveries (Figure 1) [20].

Using cyclic voltammetry (CV), Miller et al. in the 1980s developed the first controlled release mechanism for the dopamine neurotransmitter, which is physically adsorbed and cleave-bonded to the CP. The use of glutamate and ferrocyanide dopants in PPy was followed in 1984. Zinger et al. were the first to propose the use of recurring electric impulses to trigger the release of small quantities of ferrocyanide ions from the polymer in a controlled manner. The device, however, was unworkable due to the limited number of integrated molecules in the polymer (about 3.2 × 10^−8^ mol cm^−2^) [21,22,23].

**Figure 2:**
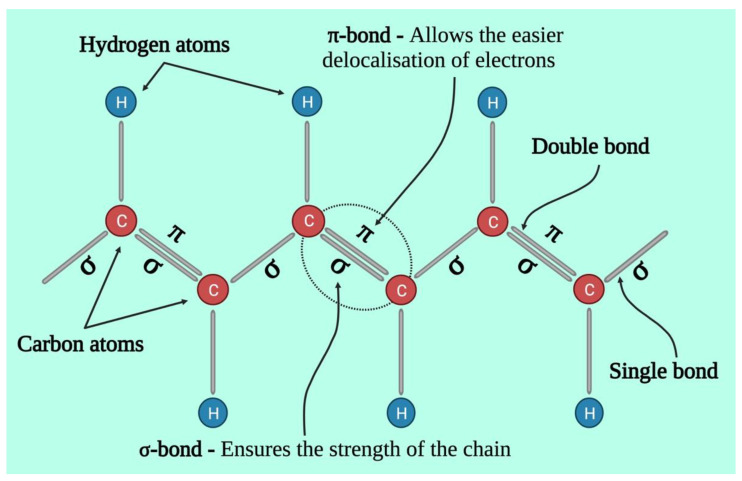
Structural representation of conductive polymer backbone.

**Figure 3:**
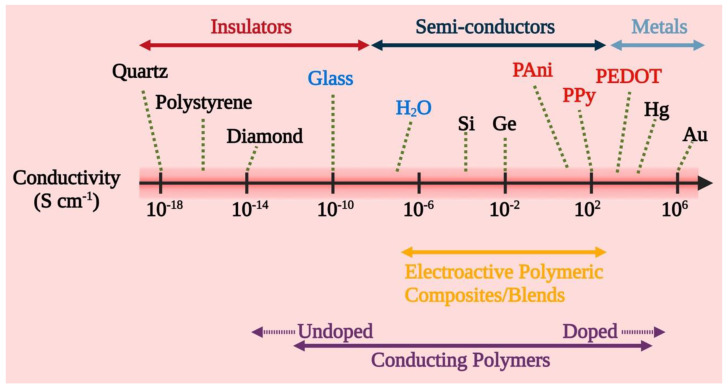
Conductivity range and conductivity-based classification of CPs and CP composites into insulators, semiconductors, and metals.

Chemically, conductive polymers are organic polymers or molecules having π-conjugated systems, and their atoms in the carbon skeleton are connected via σ-bonds as well as the extended overlapping of π-electron orbitals. Thus, neutral conductive polymers have an electronic semiconductor structure with a 1 eV energy gap between a conduction band (empty π*-band) and a valence band (completely filled π -band) (Figure 2). Doping, i.e., the withdrawal or injection of electrons onto a conjugated polymeric chain, produces intrinsic conductivity, whereas counter ions, or dopants, maintain overall electroneutrality. p-doping (oxidation) or n-doping (reduction) reactions result in the delocalization of charged structural defects of bipolarons, polarons, and solitons which act as charge carriers and are energetically located inside the energy gap band. As a result, electrochemical or chemical doping/de-doping (Table 1) [24] can be utilized for controlling the electrical conductivity of these materials across the entire range from metallic to insulating (Figure 3) [25]. Since the conductivity range can differ from less than 10^−6^ S/cm in the neutral state to more than 10^5^ S/cm in the doped state, doping significantly increases the conductivity of polymers. The conductivity of polymers is affected by the temperature, pH, polymer shape, polymer chain length, the concentration and nature of the dopant, swelling and deswelling, and applied voltage. Most of the heterocyclic polymers, for instance, PTh or PPy, use the p-doping process of conduction, which begins with the removal of one electron from the initial monomer and results in the development of an unstable radical cation called polaron. Then, from another monomer or oligomer, a second electron is withdrawn, resulting in the production of a dication, namely bipolaron. These polarons and bipolarons act as electric charge carriers that delocalize along the length of polymer chains when an electric field is applied, and their mobility along the polymeric chains generates electrical conductivity [26].

Researchers are drawn to conducting polymers because they offer a broad range of electrical conductivity similar to metals while retaining the mechanical characteristics of the polymer. To change the conductivity of a conducting polymer, researchers can alter the doping amount and utilize different dopants. Additionally, the production conditions, as well as the type of monomers and dopants utilized, affect the conductivity of conducting polymers [25]. Although conducting polymers has advanced in terms of active materials and manufacturing techniques, there are still many drawbacks and obstacles. Some polymers, for instance, are unstable and sensitive to particular conditions. Surface tension and viscosity are important factors, and additives are required to improve stability and polymer processability, which can diminish the conductivity of the polymer, particularly for drug-delivery polymers [29].

### 2.1. Types of Conducting Polymer

There are now around 25 conductive polymer systems available, including polypyrrole and polythiophene, and its derivatives like poly (3, 4-ethylene dioxythiophene), polyaniline, polythiophene-vinylene, polyacetylene, polyazulene, polyisoprene, etc., which were first developed decades ago [30]. The quest for a superior conducting polymer started because polyacetylene is difficult to produce and unstable in air. Polyheterocycles have since evolved into a class of conductive polymers with excellent conductivity and endurance. Polypyrrole, polythiophene polyaniline, and poly (3, 4-ethylenedioxythiophene) are the most promising conductive polymers for use in biomedical fields (Table 2) [31].

#### 2.1.1. Polypyrrole

Doped polypyrrole has been the most intensively studied CP for its application in biomedical due to its ease of surface modification, ease of production, and high electrical conductivity. PPy has been demonstrated to have great environmental stability and to facilitate cell adherence and growth in a wide variety of cell types [32,33,34,35,36]. Tissue engineering [34], biosensors [18], drug delivery [37], and bio actuators [38,39] are among the biomedical uses for which PPy has been investigated [40]. At room temperature, the synthesis can be easily scaled up to large amounts in water or several common organic solvents [41,42]. Depending upon the type and amount of dopant used, PPy films can have conductivities of up to ~10^3^ S cm^−1^ [31,43]. Richardson et al. employed PPy-coated electrodes to transmit charge and neurotrophins to spiral ganglion neurons (SGNs) to prevent the degeneration of SGNs caused by the usage of cochlear implant use. Cochlear implant electrodes were coated with an electrically conductive PPy/p-toluene sulfonate which contains neurotrophin-3 (PPy/pTS/NT3). In vivo investigations on guinea pigs indicated that the cochlear implant may be used to administer neurotrophic drugs to spiral ganglion neurons in a controlled and safe manner over a short period of time, as well as electrical stimulation for improved SGN preservation post-hearing loss [37].

#### 2.1.2. Polyaniline (PANI)

Polyaniline, commonly known as aniline black, was initially identified as a dye and has since been the subject of extensive research [44]. It was not until the mid-1980s that its electrical and chemical properties were documented. Polyaniline is the second most studied CP having various benefits, including a wide range of structural shapes, excellent stability, cheaper, and the potential to electrically transit between conducting and resting conditions via the doping or dedoping process [33,45,46,47,48]. The fully reduced leucoemeraldine base, half-oxidized emeraldine base, and fully oxidized pernigraniline base are the three different forms it takes depending on its oxidation level. Polyaniline emeraldine is the most stable and conductive base [9,49]. PANI is especially challenging to process because it has low solubility in most solvents [48].

In research conducted by Humpolicek and associates, both the non-conductive and conductive versions, i.e., PANI emeraldine base and PANI hydrochloride, respectively, were tested for sensitization, skin irritation, and cytotoxicity [50]. In vivo skin sensitization and irritation tests were conducted, whereas in vitro cytotoxicity tests were conducted on immortalized non-tumorigenic keratinocytes and human hepatocellular carcinoma cell lines. Both PANI base and PANI hydrochloride showed outstanding biocompatibility in terms of cutaneous sensitization and irritation, according to the findings. However, both polymers were cytotoxic, with PANI hydrochloride having higher cytotoxicity than the PANI base. Additionally, purification of these materials via the deprotonation/reprotonation cycle shows a considerable decrease in cytotoxicity, indicating that low molecular weight (MW) reaction by-products or residues, rather than polyaniline alone, are responsible for the observed cytotoxicity. Low cellular compatibility, poor processability, lack of flexibility, and non-biodegradability are the key obstacles to employing polyaniline and its derivatives in biomedical applications [46,51,52]. However, polyaniline has been studied for use in biomedical fields such as neural probes, controlled drug delivery, tissue engineering, and biosensors showing encouraging results [9,50].

#### 2.1.3. Polythiophene (PTh) and Derivatives

Polythiophenes exhibit characteristics that are similar to, and sometimes even superior to Ppy [53,54]. Neural probes, biosensors, and electroactive scaffolds for cell culture have all been investigated using polythiophene and its derivatives [55,56,57,58]. The most effective PTh derivative, Poly(3,4-ethylenedioxythiophene) (PEDOT), is employed in biotechnology and biomedicine due to its chemically stable nature and stronger electrical conductivity [59,60]. PEDOT has only recently been investigated in comparison to PPy and PANI. PEDOT’s biocompatibility has been widely documented [61,62].

PEDOT can be produced into nanofiber mats, nanofilms, and nanorod arrays, among other things. Using polystyrene sulfonic acid (PSS) and PEDOT, Mattoli et al., developed free-standing, conductive, ultra-thin nanofilms using a modified supporting layer technique. According to the study, the ratio of PEDOT: PSS nanofilms could be altered, unfolded, and folded repeatedly in water without cracking, disaggregation, or losing their conductive properties, indicating that they could be used in actuation and sensing along with other biomedical applications, such as smart substrates for simulation and cell culturing [63,64].

**Table 2:** Brief overview of conductivity and other properties of commonly conjugated CPs used in the field of biomedicine.

Type of Polymer	Type of Doping	Conductivity (S cm^−1^)	Properties	Ref.
Polypyrrole (Ppy)	p	10–7.5 × 10^3^	Easy preparation and surface modification. High electrical conductivity	[34]
Polyaniline (PANI)	n, p	30–200	Low cost. Diverse structural forms. Environmentally stable.	[65]
Polythiophene (PTh)	p	10–10^3^	Ease of preparation. Good optical properties. High electrical conductivity.	[31]
Poly(3,4-ethylene dioxythiophene) (PEDOT)	n, p	0.4–400	Transparent conductor. Electrochemically and environmentally stable	[66]

## 3. Synthesis of Conductive Polymers

Conductive polymers can be made synthetically by employing either chemical or electrochemical methods. Each method has its own advantages and limitations. Condensation polymerization (also known as step-growth polymerization) and addition polymerization are two methods of chemical synthesis (i.e., chain-growth polymerization). Small molecules, like water or hydrochloric acid, are lost during condensation polymerization. Addition polymerizations include radical, cation, and anion polymerizations, and they can be identified from one another during synthesis by the reactive end of the polymeric chain, respectively. The solution containing monomer is combined with an oxidant species, such as ammonium persulfate and ferric chloride, during chemical synthesis. This approach is preferred for commercial applications because it produces the polymer as a thick film or powder and enables bulk production [31]. Chemical synthesis allows for scale-up, which is currently not feasible with electrochemical synthesis, and also offers various pathways for the synthesis of a wide variety of conductive polymers. However, the conductivity of polymers synthesized chemically has always been lesser than that of their electrochemically produced equivalents. Additionally, it is known that the conductivity of the synthesized polymer is extremely sensitive to the type and purity of the solvent system, the relative concentration of the reagents, the oxidant, temperature, duration of reaction, rate of stirring, etc., making chemical synthesis a challenging task [67,68].

**Figure 4:**
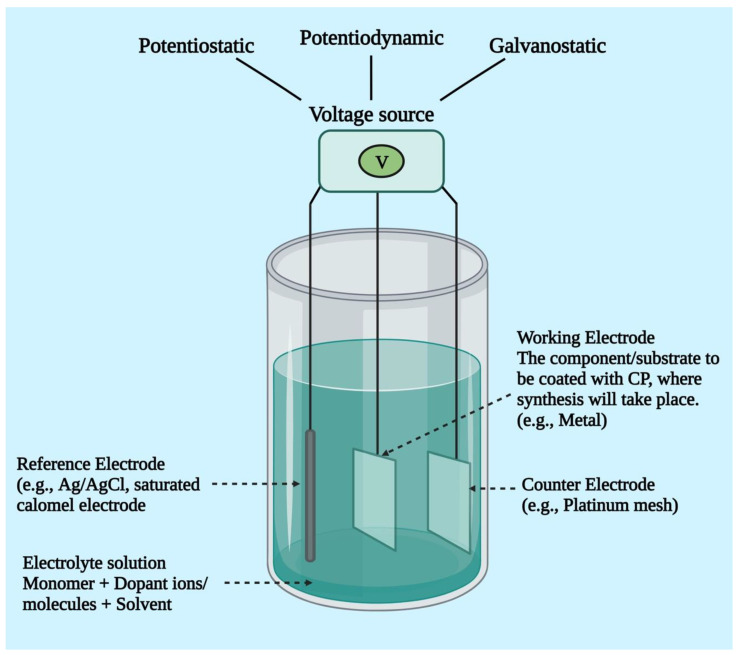
A diagrammatic representation of the electrochemical polymerization set-up.

Electrochemical synthesis is a popular technology for developing conductive polymers, particularly because it is a relatively simple technique. It takes place by passing an electrical current via electrodes positioned in a solution made up of a monomer, a doping agent, and a solvent. This technique enables the depositing of a thin polymeric film with a well-controlled shape and thickness (down to 20 nm). The monomer deposits and oxidizes on the positive electrode as a result of the electrical current, generating insoluble polymer chains. Polymers can be synthesized via electrochemical polymerization only when the monomer undergoes an oxidation reaction in the presence of an electrical potential. The major conducting polymers currently in use, i.e., PANI, Ppy, and PEDOT, all meet this requirement [69,70]. In 1968, an aqueous solution of sulfuric acid and pyrrole was subjected to oxidizing potential and precipitated on a platinum electrode, which was the first electrochemical production of CPs. Electrochemical polymerization employs a configuration of three electrodes, namely the working electrode, reference electrode, and counter electrode, in a monomer, solvent (dopant), and electrolyte solution (Figure 4). Electro-deposition takes place at the positively charged anode as current flows through the solution. Monomers are oxidized to form radical cations on the working electrode surface, which then interact with additional monomers or radical cations to form insoluble polymer chains. Key factors to be taken into consideration are electrode system, deposition charge, electrolyte, solvent system, and deposition duration and temperature. Each of these factors affects the material’s conductivity, dynamics, film morphology (such as thickness and topography), and other properties that directly affect its potential for biomedical applications. Stronger and more efficient conducting polymers are produced by using a non-nucleophilic, non-protic solvent, as protic solvents can initiate side reactions with the developing conducting polymeric chain, restricting and inhibiting chain prolongation. Three methods—potentiostatic, potentiodynamic, and galvanostatic methods—can be used to conduct electrochemical synthesis (Figure 4). In the case of potentiostatic polymerization, the current varies, whereas the electrical potential of the electrodes is regulated. This process is appropriate for the production of biosensors since it maintains the integrity of the element that will be coated. A coulometer is required to regulate the quantity of polymer being deposited because the current might vary based on a number of variables (such as the material of the electrode and plating procedures) [69,71]. The potential of polymerization is cycled between a high and low potential limitation during potentiodynamic deposition. Due to this, the polymer is deposited in layers, each of which becomes active electrically until the next polymer is in the process of being synthesized [72,73,74]. During galvanostatic polymerization, the electrical current rather than the voltage is regulated. This indicates that the rate of polymer deposition is continuous and precisely controllable [69,71].

The main difference between chemical and electrochemical conducting polymer synthesis is that chemical polymerization frequently results in powders or very thick films, whereas electrochemical synthesis can generate very thin films of conductive polymers on the scale of 20 nm. All conductive polymers can be chemically synthesized; however, electrochemical synthesis can only be accomplished when the monomer undergoes oxidization in the presence of a voltage that produces reactive radical ion species for polymerization. Conventional conductive polymers, such as PTh, PANI, PPy, and PEDOT, can be synthesized either by chemical or electrochemical methods; however, numerous innovative conductive polymers with altered monomers can only be polymerized chemically [75,76].

## 4. Conductive Polymer Composites

Traditional conductive polymers, such as PEDOT and Ppy, provide excellent electrical conductivity for a wide array of applications. Conversely, their processability, biocompatibility, and mechanical properties are frequently inadequate. As a result, polymers that are biocompatible with dispersed conductive fillers, including carbon nanotubes (CNTs), graphene, and metallic NPs, are receiving more attention [77,78].

### 4.1. Types of Conductive Polymer Composites

#### 4.1.1. Conjugated CPs Composites

An efficient way of enhancing the mechanical characteristics of conductive polymers is to synthesize composites and blends with the help of different polymers that offer superior mechanical characteristics for the proposed applications. Additionally, the composites of conductive polymers with improved biocompatibility and mechanical properties can be synthesized using large-molecule doping. However, these methods or pathways might interfere with electron conjugation within the conductive polymer because insulating molecules exist [79]. Ma et al. developed nerve conduits synthetically by dip-coating them in a composite solution of polypyrrole/poly (D, L-lactic acid) (PDLLA) made by emulsion polymerizing PPy in PDLLA solution. The oxidative polymerization was started with an aqueous FeCl_3_ solution [80]. In vitro cell compatibility was assessed using PC12 cells, and after being stimulated with 100 mV for two hours, they formed longer and more abundant neurites on composite conduits than on PDLLA conduits. Additionally, nerve conduits that bridged a 10 mm gap in the sciatic nerve of rats were developed using the 5% PPy/PDLLA composite. After six months, the rats who had received the conduits of PPy/PDLLA demonstrated functional capacity that was much superior to PDLLA conduits and comparable to the gold standard autologous nerve transplant. The scientists hypothesized that the synthesized conduit could be employed to regenerate nerve tissue while avoiding the limitations of autologous grafts, such as multiple operation sites, donor sites, probable mismatch, and limited donor sources.

In another study, Schmidt and co-workers synthesized biomaterials for wound healing and tissue engineering by synthesizing conducting composites of Ppy using a dopant, hyaluronic acid (HA). These electrically conducting Ppy films containing HA sustained the dopant HA on the surface of the films in vitro for several days and boosted vascularization in vivo, making them potential candidates for wound-healing and tissue engineering applications that benefit from both increased vascularization and electrical stimulation [81].

#### 4.1.2. Non-Conjugated CPs Composites

By incorporating conducting fillers into insulating polymers, electrical conductivity can be increased while keeping the polymeric properties [82]. As fillers, carbon fiber, silver, carbon black, and other metallic particles are frequently employed. Recently, nanoscale conducting fillers, namely graphene, metal nanoparticles, and carbon nanotubes (CNTs), have gained a lot of attention and are actively being investigated for the production of conductive composites based on polymers [83,84,85,86,87,88,89,90,91]. The development of conducting channels of fillers within the matrix of polymer gives these composites their conductivity. Many factors impact the design of conductive paths, including intrinsic qualities, dispersion, abundance, and shape of the nanofillers. Furthermore, the interactions between the matrix and the filler are crucial in shaping the nanocomposite’s electrical characteristics. Consequently, it is essential to select a suitable composite preparation technique that ensures the necessary distribution of filler [92].

Graphene is a two-dimensional (2D) monolayer of carbon with sp2 hybridization that resembles a honeycomb hive and has specific electrical conductivity, surface area, and higher mechanical strength [93,94]. Graphene-based polymer composites outperform pristine polymers in regard to their electrical, mechanical, electrical, and thermal characteristics [90,95]. Due to graphene’s hydrophobic nature, stable dispersions in polar liquids require the use of appropriate surfactants [96,97].

Similar to graphene, graphene oxide (GO) has polar functional groups that contain oxygen which help enhance biocompatibility, polar solvent compatibility, and polymer matrix compatibility [88,97,98,99]. This is of particular interest in biomedical fields because of their significant characteristics, such as excellent electrical conductivity, high surface-to-volume ratio, thermal stability, chemical inertness, and solubility in aqueous media [100]. The cellular interactions are significantly influenced by the surface chemistry and charge of GO. Since oxygenated functional groups on GO have a high negative charge density, there may be electrostatic interactions occurring between GO and biological membranes. Moreover, they have extremely high mechanical strength and rigidity, enhanced conductivity, and excellent optical transparency; these materials are generally regarded as potential candidates for biomedical applications [101,102]. Cell adhesion at the surface of the biomaterial is also improved by using hydrophilic graphene-based fillers like GO [97]. Carbon nanotubes are another type of carbon-based filler that can be utilized to make electrically conducting nanocomposites. Carbon nanotubes have an excellent electrical conductivity of more than 10^3^ S cm^−1^ and a higher aspect ratio of 100–1000 μm in length for both single- and multi-walled carbon nanotubes [103,104,105]. Metal nanoparticles, in addition to carbon-based conductive fillers, have been investigated for imparting conductivity to non-conjugated insulating polymers.

### 4.2. Synthesis of CP Composites

#### 4.2.1. Solution Mixing

**Figure 5:**
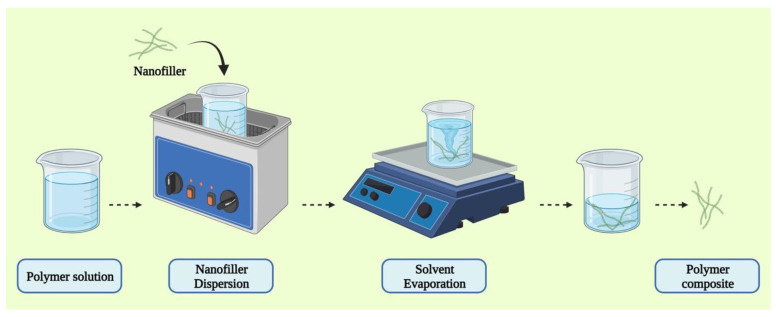
Schematic representation of solution mixing method of CP composites synthesis.

Solution mixing is one of the most frequently used approaches for CNT and graphene-based polymer composites because it allows for the dispersion of nanotubes or the separation of graphene sheets [99,104,105,106,107,108]. A polymer solution is generated using this process, and the nanofiller is dispersed separately using sonication in an ideal solvent medium [104]. This method utilizes surface-modified nanotubes to produce a metastable dispersion in CNT/polymer composites. After the filler has been dispersed in the solvent, the previously dispersed polymer in the same solvent is added to this dispersion containing the filler enabling the adherence of the filler to the polymer. The solvent is then removed by the process of evaporation (Figure 5). This approach has been used to make composites utilizing both organic solvents and water.

#### 4.2.2. In Situ Method

The filler is initially swelled in a liquid monomer before being polymerized in situ. After that, an appropriate initiator is dispersed, and polymerization is started via radiation or heat. Polymer and CNT composites are primarily produced by the process of in situ polymerization because it has the advantage of generating a covalent link between the matrix and the CNT. Polymeric chains on the tube’s surface aid dispersion while also creating a strong contact. This method enables the synthesis of nanotube composites having a high-loading capacity that otherwise would be diluted by different methods [91,104].

#### 4.2.3. Melt Processing

Melt processing, also known as melt blending, has gained popularity because of its absence of solvents. In this procedure, the polymer matrix and additional nanofillers are mixed while they are still molten [109]. Using traditional processes such as extrusion and injection molding, the filler is mechanically combined with the thermoplastic polymer at higher temperatures [110]. Nanocomposites are produced by the intercalation of polymer chains between the filler. During this process, the conformational entropy of the polymer chains is significantly reduced [104]. Owing to its simplicity and speed, melt blending is the preferred choice for industrial production. Further, this is the most preferred method of polymer processing that cannot be mixed in a solution or polymerized in situ [91].

#### 4.2.4. Latex Technology

Another approach for manufacturing CNT- and graphene-based polymer composites is latex technology, which offers benefits such as uniformly dissolved fillers in the polymeric matrix, process scaling, and ease of processability. This method can be applied to any filler that can be dispersed to produce a dispersion of aqueous colloidal solution and any polymer that can be produced artificially as polymer latex or via emulsion polymerization. The latex method enables the synthesis of a three-dimensional (3D) structure of filler within the matrix of the polymer, as well as the incorporation of nanofillers into the viscous matrix.

The three basic stages of latex technology are preparing aqueous colloidal dispersion of nanofillers, generation of a two-component colloidal solution by combining the colloidal mixture with polymer latex, and lastly, lyophilization to yield a composite. Various nanocomposites of polystyrene (PS)-graphene and polymer-CNT have been successfully synthesized using this technique. The prepared graphene-PS nanocomposites had maximum conductivity of 0.15 S cm^−1^ and percolation thresholds of 0.8 wt.% for graphene loadings of 2 wt.%. Controlled clustering of the graphene filler was also shown to favor the reduction of the percolation threshold in this study [90,111].

## 5. Importance of Conductive Polymers in Drug Delivery

Chemical substances must be delivered in a regulated manner in many branches of science, including medicine, pharmaceuticals, and agriculture, which has been a major hurdle [112]. The utilization of CPs as a substrate for programmable DDS appears to be overcoming this major obstacle. CPs have gained significant interest due to their lowering (negative) electrical potential that can be used to control the expulsion of molecules bound in polymers due to the process of doping [112,113,114,115]. Conductive polymers can be made permeable, and their delocalized charge carriers facilitate the dispersion of linked molecules, making them suitable for the purpose of drug release [112]. Additionally, CPs enable external control of electrical stimulation duration and intensity, which is advantageous in biological applications. As a result, CPs have a variety of capabilities that are beneficial for diagnoses and treatments of damaged body parts, such as alteration of cellular response to physical, electrical, or optical characteristics; easy functionalization with bioactive molecules; dimensional modification upon reduction and oxidation; charge transfer from an electrode to ions in living tissue; and encapsulation and release of biomolecules and drugs. Another important benefit of their usage in bioengineering is their in vitro and in vivo biocompatibility [116].

In studies, many therapeutic drugs have been encapsulated using various techniques, for instance, electro-polymerization, graft co-polymerization, and electrochemical synthesis, releasing efficiently from these polymers through various electrical stimulations, such as CV-triggered release, including dexamethasone, dopamine, 2-Ethylhexyl phosphate, nerve growth factor (NGF), heparin, and naproxen [114,115,117,118,119,120]. Neurotrophin-3 [121,122], NGF, and brain-derived neurotrophic factor (BDNF) were efficiently released using PPy, resulting in neuronal development and differentiation [36,123,124,125]. In one experiment, a hydrogel made of poly (vinyl alcohol) produced on a PPy film released heparin, whereas, in another, heparin was exposed on the surface after being exposed to an electrical potential between 0.4 and 0.7 V for 90 s [123,124].

### 5.1. Biocompatibility

Validating the materials’ biocompatibility is critical when they are destined to be incorporated into biological systems. The applicability of the materials depends on the synthesis route of the bioorganic materials as well as the general characteristics of the conjugated polymers, like their surface charges, acidity, or chemical composition. Depending on the material and synthesis method used, the polymer film might have residues such as excess doping ions, detergents, monomers, or solvents. These substances can be harmful to cells if they leak out of the film while an experiment is being conducted. The surface topography can also influence the interaction between the surface and the cell [126]. For many biomedical applications, a good reaction of cells to the biomaterial is required [127,128]. Many CPs, such as PANI, PTh, PPy, and polyethyleneimine (PEI), have been demonstrated to facilitate the growth of a diverse spectrum of cell types, which is essential [9,129,130]. In addition, the biocompatibility of CPs can be enhanced by adding biocompatible substances, side chains, and segments to the polymer if necessary [121]. CPs allow for the development of electrode materials that are more chemically and mechanically similar to brain tissue than silicon or metals, implying that they may have a better chance of forming a stable interface with neurons. In vitro and in vivo biological tests are critical components of biomaterial development. Many have demonstrated that critical cell types may be cultured on top of PPy and PEDOT films [131].

### 5.2. Biodegradability

Many CPs (such as PTh and PPy) are not naturally biodegradable [7,9,132]; however, they can be made biodegradable using a variety of techniques [38,46,133]. The first method involves combining conductive polymers with a biodegradable polymer to synthesize a composite [134,135]. Erodible polypyrrole nanoparticle-polylactide (PLA) composites and PPy-PLLA composites, for example, have been effectively manufactured [135,136]. Despite the fact that this technique combines the advantages of the two polymers, it fails to address the problem of eliminating the CP from the body after the degradable polymer has been removed [134,135,137]. This approach, on the other hand, has the advantage of being able to control the conductance and rate of deterioration by selecting the proper blend of the two polymers [135]. The second method involves altering the conductive polymer itself. It has been reported that the addition of hydrolyzable (butyric ester) or ionizable (butyric acid) side chain groups to the backbone of PPy make it degradable. The rate of deterioration can be controlled by selecting the appropriate number of these groups [8,112,138]. The third approach involved an electrochemical synthesis of tiny chains of PPy that, by virtue of their small size, are capable of gradual erosion and renal clearance [138].

## 6. Functionality for a Specific Application

Most conductive polymers offer a wide number of key benefits in the biomedical field, such as the potential for charge transfer from a biochemical reaction, biocompatibility, the ability to encapsulate and controllably release biomolecules (i.e., reversible doping), and the ability to modify the chemical, physical, electrical, and other characteristics of the CPs to better adapt to the nature of the particular application. Numerous biological applications, such as tissue engineering scaffolds, neural probes, biosensors, bio-actuators, and DDS, benefit from these distinctive properties [135].

The material characteristics of conductive polymers, such as porosity, roughness, conductivity, hydrophobicity, and degradability, as well as the binding of biological molecules, which makes CPs attractive for various biomedical applications, can be optimized in the following ways:

### 6.1. Adsorption

**Figure 6:**
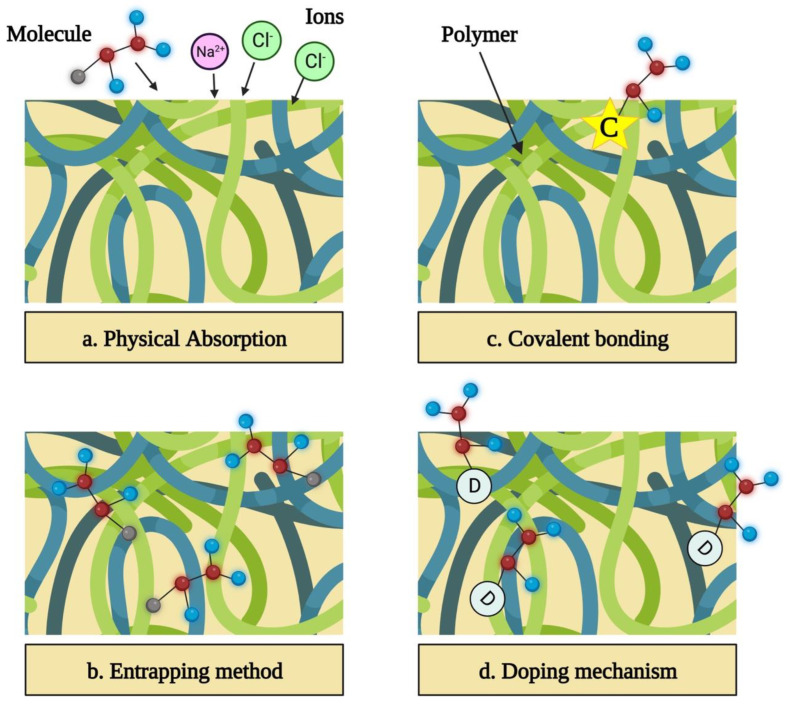
Functionalization of conductive polymers for specific applications.

In this method, a sample that contains a functionalizing compound is kept in close vicinity of a polymer that has already been synthesized. The biomolecule is physically absorbed as a result of the static interactions between the matrix of the polymer and the charge of the molecule (Figure 6a) [139].

### 6.2. Entrapment of Molecule within the Polymer

This can be accomplished by combining the solvent, dopant, and monomer with the functionalizing molecule prior to synthesis. Electrochemical polymerization involves the integration of functionalizing molecules near the electrode into the developing polymer [140]. Since big molecules, like DNA and enzymes, are unable to escape the polymer once entrapped, this approach is largely used to bind them. Adsorption and entrapment are basic strategies that allow biomolecules to be incorporated without undergoing a chemical process that can alter their activity (Figure 6b) [141,142].

### 6.3. Covalent Bonding

Covalently binding the molecule to the polymer’s monomer increases the polymer’s long-term stability and guarantees that the biomolecules are firmly linked and will not be released (Figure 6c) [143].

### 6.4. Exploiting

This allows various biomolecules to bind as long as they are charged [115,122,144,145]. Doping has already been used to successfully bind collagen, growth factors, heparin, ATP, and chitosan in conductive polymers. Unfortunately, doping bioactive compounds allows only a limited percentage of the molecules to be bonded, and it has a bigger detrimental impact on the conductivity of the polymer than the covalent bonding (Figure 6d) [9,146,147,148].

## 7. Factors Influencing the Drug Delivery

### 7.1. Morphology and Shape

By modifying the morphological structure, the functions of CPs can also be controlled. Therefore, it is essential to establish a connection between morphology structure and functionality. This section describes the merits and physicochemical characteristics of various morphology, such as porous-like, sphere-like, and rod-/fiber-/wire-like [149].

#### 7.1.1. Porous-like Structures

The ability of porous structures to incorporate several chemical functions within their porous network or on their surface has attracted a lot of interest. Due to their capacity to have both porous qualities and intrinsic polymer materials, designing porous structures at the micro- and nanoscale levels has long been a crucial topic. These structures have well-defined porosity and a greater surface area per unit volume. Moreover, the pores’ diameters can be altered to achieve particular features. Extensive research is also being conducted on a number of significant characteristics, such as size, geometry, and functioning of the pore, for applications employing CPs. Due to their higher specific surface area than bulk materials, porous electrode materials have recently attracted a lot of attention. Porous substances with larger pore diameters offer enhanced electrolyte accessibility, which facilitates the transport of ions and can significantly increase rate capability [150]. By utilizing electrospun PE44 fibers as a temporary template to synthesize the hollow PEDOT nanotubes, Estrany et al. demonstrated that it was easier to control the inside/outside diameter as well as the length of the nanotubes by modifying the template’s morphology. In particular, the hollow interior of the tubes would increase the surface area exposed for reactions, resulting in enhanced performances [151]. In another study, Olejnik and co-workers developed a PANI-based nanotube and multilayered fullerene-based nanocomposites, which exhibit 946 F g^−1^ of specific capacitance at a scan rate of 1 mV s^−1^ and have a brush-like appearance. Due to both nanostructures’ high conductivity and ordered brush-like structures, which have extraordinarily large porosities, the nanocomposite has greater specific capacitance values. In particular, “conductive” channels were developed in which charge transfer is facilitated by interactions between the multilayered fullerenes and p-electrons of the PANI quinonoid and aromatic structures [152].

#### 7.1.2. Sphere-like Structures

Nanospheres generally stand out from macroscopic materials due to their smaller size of particles, spherical nature, ordered atom arrangement within the particle, and larger specific surface area. In many disciplines, including the biomedical field, sphere-shaped CPs are frequently used. Nanospheres’ physical and chemical features were primarily influenced by their morphology, surface characteristics, and size. Further, to enhance their effectiveness, nanospheres with a variety of morphological features, such as hollow, urchin-shaped, and porous nanospheres, have been developed [149]. Especially in the area of electrochemical energy storage, porous CP nanospheres have proven to be promising [153]. Urchin-shaped CP nanospheres have a large specific surface area, which offers them enhanced electroresponsiveness, catalytic performance, and electroactivity, similar to porous nanospheres [154]. However, hollow nanospheres possess distinct pore characteristics due to the presence of hollow space. These properties mostly depend on the larger specific surface area, lower density, and materials’ functionality. Because of their multiple desirable features, these materials have attracted a lot of attention [155]. For instance, Hu et al. synthesized PANI nanospheres that resembled urchins and had a greater specific surface area of 267 m^2^ g^−1^, and their specific capacitance was 435 F g^−1^ (10 mV s^−1^) [156]. The varied morphology and nanostructure of CPs are related to the ionic diffusion pathway when the Faradaic reaction takes place at the interface of the PANI electrode/electrolyte, which significantly influences the performance of the capacitances. Another study carried out by Choi and co-workers reported that hollow PANI nanospheres significantly improved electrorheological functions when compared to the urchin-like PANI nanospheres because their hollow structure had a reduced diffusion length, which facilitated the transfer of electrons and stresses. Such a hollow structure can also be altered further to offer novel functionality [155].

#### 7.1.3. Rod-/Fiber-/Wire-like Structures

1-dimensional (1D) CPs have a larger aspect ratio than nanospheres, which allows them to transmit electrical carriers along a single, controlled direction more effectively. Due to their distinctive electrical, optical, mechanical, and anisotropic features, they are used widely in various applications. The synthesis of CPs that resemble rods, fibers, and wires has received a lot of attention from researchers in recent years [157]. By modulating morphology, CPs possessing short-rod nanostructures are significantly employed in many different fields. For instance, by chemically oxidizing hexagonal spiral prismatic IC-Fe(II) chelate micelles, Hu et al. synthesized nanorods made of Ppy that have hexagonal spiral prism shapes [158]. These nanorods have a conductivity of 1.6 S cm^−1^, which is three orders of magnitude higher than that of Ppy film. The physicochemical characteristics of nanorods are more strongly influenced by their lengths and widths. It is important to take into account the size effect when designing CPs because the electrical properties depend on the diameter. As a result, wire- and fiber-like CPs are synthesized and are employed most frequently in different electronic devices. Due to their enormous length-to-width ratio, CP nanowires exhibit distinctive optical and electrical characteristics. The 1D structure encourages more efficient doping and boosts the interactions between the intra- and inter-chain to increase the level of crystallinity, which leads to enhanced conductivity. Due to the precise ordering of the polymer chains, single-crystal CPs have the maximum conductivity when compared to amorphous CPs [149]. For example, Sung and co-workers synthesized nanowires comprising single-crystal PEDOT through vapor phase polymerization of 3,4-ethylenedioxythiophene (EDOT). The single-crystal PEDOT nanowires have an ultrahigh conductivity of up to 8.797 S cm1, which demonstrates promising application in organic nanowires. The single-crystal PEDOT nanowires’ significant increase in mobility raises the possibility that their ultra-high conductivity is mostly due to the narrow stacking distance of p-p and their extremely crystalline structure [159].

### 7.2. Density and Thickness of the Polymer

A CP film’s thickness during synthesis is influenced by the degree of polymerization and the polymer’s density [160]. Several studies have demonstrated that there is enhanced drug release as the thickness of the polymer increases [161,162]. The majority of this research compared films produced using identical synthesis conditions. The amount of drug correlated with the amount of polymer indicates that there is more availability of the drug for release, presuming that the drug loading weight percentage remains relatively constant. Relatively thin films release a higher proportion of the encapsulated drug than thicker films, although the level of drug release does not rise linearly with the increasing thickness of the film. This might be because thick films are less electroactive and because variations in diffusion coefficient occur as film thickness changes [163].

### 7.3. Electrochemical Parameters

In an effort to regulate the release of medications, CPs can receive an array of electrical stimulations. Step potential is the instantaneous switching of the potential between two preset potentials. CV entails sweeping the potential at a predetermined rate between two limits. The potential limitations can be adjusted to take advantage of the polymer’s various redox states. A charged bioactive agent will alternately either encounter or lack the attraction forces when the CP redox status changes. The CP’s ability to actuate when it switches between redox states may have an impact on the transportation of drugs. Researchers have studied the step potential versus CV stimulations for drug release and concluded that the method of choice for the releasing ions, CV appears to be more effective [1,114,119,162,164]. Thompson et al. discovered that CV released NT-3^+^ more quickly than both current pulses or rapidly alternating potential steps, and delamination of Ppy coating from the electrode was observed. Longer contact time with the underlying substrate was maintained upon application of pulsed potential or pulsed current [161]. Wadhwa et al. further observed that CV was the more effective method for stimulating drug release, although, after 30 cycles of CV, cracks began to emerge on the CP [114].

### 7.4. Drug Release Medium

Drug release from CPs into different solvents has been the subject of numerous research. It has been demonstrated that CP properties and drug release are influenced by media qualities, such as hydrophobicity, pH, polarity, and ionic strength. For instance, both cationic and anionic movement is involved in the transport of ions at neutral pH, whereas anion movement predominates below pH 3–4. However, the media utilized for evaluating drug release should replicate the intended environment where the device will be utilized to establish a correlation between in vitro and in vivo release. Implants that interact with the extracellular fluid should release their drug at pH 7.4. Therefore, it would be necessary to take pH into account if CPs are designed to be employed in a wide range of environments, such as the rumen of an animal [165,166,167].

## 8. Applications

Conductive polymers have similar electrical and optical characteristics to inorganic semiconductors and metals, as well as the desirable qualities associated with traditional polymers, such as ease of processing and synthesis [168]. These materials have a multitude of applicability in the field of microelectronics, such as light-emitting diodes, batteries, electrochromic displays, and photovoltaics. Recently these polymers are also being utilized in the biomedical field. Research of conductive polymers in the biomedical field significantly increased in the 1980s when it was discovered that these materials were suitable for many biomolecules, including those employed in biosensors. The ability of CPs to alter biological processes, such as DNA synthesis, protein release, cell adhesion, and migration, through electrical stimulation had already been established by the mid-1990s [169,170,171,172]. Numerous research papers focused on electrically responsive cells, such as those in the heart, bone, muscle, and nerve. The majority of CPs offer several key benefits for biomedical applications, such as the capacity to encapsulate and release biological molecules under controlled conditions (also known as reversible doping), biocompatibility, the potential charge transfer from a biochemical reaction, and the capability to modify the physical, chemical, electrical, and other characteristics of the conducting polymers, to better suit the needs of the particular application. Numerous biological applications, including drug-delivery systems, bio-actuators, biosensors, neural probes, and tissue-engineering scaffolds, benefit from these distinctive properties (Table 3) [135].

In addition to CPs, other electroactive materials have been employed in biological applications [16]. For instance, nerve conduits have been developed utilizing electrets and piezoelectric materials, which generate transitory electrical charges in response to mechanical deformation. Sensors and neural probes are manufactured using metals and semiconductors, for example, silicon, iridium, and gold. However, compared to these materials, CPs have a multitude of benefits. Comparatively, CPs are less expensive, easier to fabricate, and more adaptable because of the wide variety of compounds that can be either entrapped in them or employed as dopants, easily altering their properties. Furthermore, in the case of tissue engineering, unlike electrets, CPs control the duration and intensity of electrical stimulation [173,174]. Moreover, CPs can be modified to provide substrates with a large surface area, essential for lowering neural probes’ impedance. Particularly, the surface area of CPs can be up to 50 times more than that of bare iridium electrodes [175]. Moreover, CPs can be accurately coated on metallic electrodes for their application in biosensors and can closely interact with proteins to generate a transduction mechanism that is more efficient.

**Table 3:** Biomedical applications of CPs.

Type of Application	Description	Polymers Currently Explored	Merits	Drawbacks	Ref.
Drug Delivery	Devices utilized for storing and regulatomg drug release.	Ppy PEDOT	Controlled release with reduction. Ability to encapsulate biomolecules.	Rapid release. Entrapped proteins can get denatured by hydrophobicity.	[176,177]
Biosensors	Devices containing biomolecules as sensing substances that are coupled with electrical transducers	Ppy PANI PTh	Possible surface modification. Ability to encapsulate biomolecules in films. Electrochemical synthesis on metallic electrodes. Efficient electric charge transfer	Hydrophobicity can destroy the encapsulated proteins. Diffusion barriers for entrapped enzymes.	[178,179,180]
Bio-actuators	Devices for producing mechanical power that can be utilized as “artificial muscle”-type actuators.	Ppy PANI Polymer-carbon nanotube composites	Good conductivity. Biocompatible. Light in weight Can control dopant uptake/release. Works at body temperature and fluids.	Response limited by ion mobility. Delamination of conductive polymeric films. Short-term redox stability.	[181,182,183]
Tissue engineering	Biodegradable and biocompatible scaffolds of tissues containing stimuli to enhance regeneration of tissues.	Ppy PANI PTh and derivatives Novel conductive polymers	Potential modification to incorporate chemical stimuli. Excellent conductivity Biocompatible	Hydrophobicity Not highly porous. Not biodegradable.	[184,185,186,187]
Neural probes	Implantable electrodes in brain for stimulating and recording neurons.	Ppy PEDOT	Increased surface area (decreased impedance). Electrochemical polymerization on metal electrodes. Good stability and conductivity. Biocompatible.	Reduced electrical contact at interface.	[188,189]

PPy: polypyrrole; PANI: polyaniline; PTh: polythiophene; PEDOT: Poly(3,4-ethylenedioxythiophene).

### 8.1. For Drug Delivery

#### 8.1.1. Polymeric Films

The simplest and most common forms of CPs are thin films generated electrochemically, and electropolymerization of CPs is a well-established technique that may tailor the thickness of film while adding bioactive substances as doping agents. As a result, electrochemically produced polymer films are extensively used for drug release on demand. The incorporation of drugs with various chemical properties, such as methotrexate [190], dexamethasone [145], heparin [177], sulfosalicylic acid [191], chlorpromazine [192], and risperidone [193], into conductive polymeric films to achieve their controlled and regulated release has been demonstrated in prior investigations. Films made of PEDOT, PPy, poly (N-methyl pyrrole) (PNMPy), oligoaniline-PEG, oligoaniline, oligoaniline-alanine, and oligolaniline-PCL are a few examples [194]. According to Krukiewicz et al., biologically active molecule-containing PEDOT films exhibit great cytotoxic effects against MCF-7 and KB cell lines, and the death of cells increases rapidly following the release of botulin which was aided by the potential difference. As a result, these have more potential for using local chemotherapy [195]. A recent discovery revealed the recording of brain activity by electrodes implanted in rats’ hippocampus and the prevention of local inflammatory reaction by CV-triggered release of drugs weekly. CV is an electrochemical method used to measure the current that generates in an electrochemical cell when the voltage exceeds the level indicated by the Nernst equation. A working electrode’s potential is cycled during CV, and the resulting current is then measured. Dexamethasone, an anti-inflammatory medication, is controlled-released from PEDOT film. After a 12-week evaluation period, test electrodes that released the medication had closer-spaced neurons than control electrodes [196].

#### 8.1.2. Polymeric Nanoparticles

According to previous research, drugs with various characteristics are released from thin polymeric films that are produced by an electrochemical process. The focus has moved in recent years to developing nanostructured films with improved drug loading. When compared to conventional flat films, nanoporous films made with colloidal templates and nanowire-based films made with anodic aluminum oxide templates showed a tenfold increase in the loading capacity of the drug and release of the drug per stimulation. Nanoparticles provide for easier scaling, processing, and drug loading when compared to films. Samanta et al. have noted a variable drug release profile based on the oxidizing agents (ferric chloride, chloroauric acid, and hydrogen peroxide) utilized in the synthesis of films made from polypyrrole nanoparticles. FeCl_3_ and HAuCl_4_ are metal-based oxidizing agents that produce greater drug release per stimulus. Additionally, it was shown that when FeCl_3_ is employed as the oxidizing molecule, drugs can be released at voltages as low as −0.05V, which is nearly an order of magnitude lower than commonly utilized voltages [197].

An innovative electric and temperature dual-stimuli responsive NP system for the programmed delivery of drugs has been described by Ge et al. With the use of a temperature-sensitive hydrogel (PLGA-PEG-PLGA), conducting polymer (polypyrrole) NPs that were fluorescein and daunorubicin-loaded were localized in vivo under the skin. They have shown that the introduction of a weak, external DC electric field regulates the drug release from the conductive NPs. This approach shows an excellent temporal, spatial, and dosage control release profile that is externally controlled. Moreover, it provides a novel interactive drug delivery method [198].

By combining three-layered films made of different CPs, such as PNCPy, PNMPy, or PEDOT, and by using electrochemical synthesis techniques, Fabregat G et al. developed electrodes for the specific voltammetric analysis of mixtures for dopamine with uric and ascorbic acids as well as samples of human urine with real interferents. According to voltammetric analyses of mixtures of solutions, electrodes composed of alternating internal and external layers of PEDOT, and an intermediate layer made of poly (N-methyl pyrrole) exhibited high sensitivity and resolution. Additionally, after coating the surface of three-layered electrodes with gold nanoparticles (AuNPs), their sensitivity rose marginally [199].

Hyaluronic acid (HA) was used as a stabilizer during the effective synthesis of PEDOT nanoparticles by Winter et al. using an oxidative mini-emulsion polymerization process. The hydrophilicity and bioactivity of nanoparticles were enhanced by this technique. It was shown that nanoparticles exhibited conductivities up to ten times larger than pure PEDOT:PSS with higher oxidant addition. Conductive polymers’ potential for adaptable biomedical applications, such as cardiac patches, nerve conduits, and tissue scaffolds, was enhanced by this simple-to-manufacture method with improved characteristics [200].

Insulin, a high molecular weight polypeptide, was controllably released by Nassab et al. using conductive polymer nanoparticulate backbones. For modeling the interactions between the polymer scaffold and insulin, a straightforward Langmuir-type adsorption model was employed. By altering the ratio of NPs to insulin, they calculated the percentage of drug loading to be as high as 51 wt.%. The activity of the electrically stimulated insulin release was confirmed using an in vivo mice model [201]. A brief summary of the synthesis of electrosensitive nanoparticles, as well as electro-stimulated release obtained from the conductive polymer-based NPs, is depicted in Table 4.

#### 8.1.3. Polymeric Nanotubes, Nanowires, and Fibers

Martin et al. [176] reported on the production of conducting nanotubes employing PLGA or PLA nanofibers as hard templates. After electrochemically depositing intrinsically conductive polymers (ICPs) around the electro-spun nanofibers, PLGA or PLA with Dex dissolved in chloroform was electro-spun onto a probe’s surface. Delivery of the drug was accomplished by either regulating the PLA/PLGA’s degradation or by using an electrical field to actively act on the ICP. Diclofenac was added to bacterial cellulose (BC) microfibers by Chen et al. [206], who then covered them with a PEDOT shell. PEDOT contracted and migrated in response to electrical stimulation, exerting pressure on the BC microfiber and accelerating the release of the therapeutics. Similar to this, ICPs were used to release the active moieties from electro-spun PLA fibers that were loaded with curcumin and PEDOT NPs [207].

PPy has been extensively used to study nanowires, which are made up of elongated nanotubes entwined in a mesh. They have been prepared using a variety of strategies, including those that rely on the use of functional compounds [208,209,210], seeded growth [211], and interfacial polymerization [212]. Although there is a lot of research on these nanostructures’ capabilities in energy storage and biosensing [213], there are less data on how they could be used as DDSs. ATP was employed by Ru et al. as a model drug delivery agent and morphology-directing agent [214]. Surprisingly, the data revealed that after 45 h of electrical stimulation, there was a substantial release difference between the polypyrrole nanowires (90%) and traditional polypyrrole morphologies (53%). Electrochemical impedance spectroscopy assays supported the observation made by cyclic voltammetry that polypyrrole nanowires are significantly more electroactive than traditional polypyrrole forms and that the material resistance is significantly lower.

To boost the efficiency of drug encapsulation, polymer nanowires, fibers, and nanotubes are fabricated, including diclofenac, ATP, dexamethasone, doxorubicin, ciprofloxacin, curcumin, etc. [215,216,217,218,219,220]. This results in a greater surface-to-volume ratio. Additionally, it is frequently essential to combine intrinsically conductive polymers with other polymers, such as PLGA or PLA, in order to build such structures, giving the material improved features like increased flexibility and mechanical stability [16].

### 8.2. In Biosensors

Biosensors are now of great interest to CPs. Biosensors are analytical devices for the detection of analytes. A transducer and a biological sensing element are the typical components of a biosensor. The sensing substance reacts with the target analyte to produce a signal chemically which is transmitted to the transducer, which finally converts the input into an electrical signal. Antibodies, enzymes, DNA probes, and cell receptors that interact with a particular analyte might serve as the biological sensing component [18,221]. CPs are often utilized as transducers to combine signals from biological sensing components (Table 5).

Biosensors are capable of detecting a wide range of biological molecules by encapsulating different biological sensing substances into the CPs. Due to its environmental resilience and high electrical conductivity, PPy has been thoroughly researched for biosensor applications. Additionally, it is simple to synthesize via electrochemical or chemical processes [232,233]. In order to detect the IgG antigen of a rabbit, Gooding et al. reported a label-free immunosensor using an anti-rabbit IgG antibody at the surface of the polypyrrole [234]. The detected signals were made evident when the measured by ion flux inside and outside of the membrane of CP varied in response to the antigen’s interaction with the polymer that had been altered by the antibody.

Gao et al. developed biosensors for the detection of glucose using a mixture of Ppy and CNTs. To create a huge surface area, they employed substrate materials made of aligned CNTs. To coat the CNTs, PPy was electropolymerized. During the polymerization procedure, an enzyme called glucose oxidase, a biological sensor for glucose, was combined with the electrolyte solution and monomers. The glucose oxidase was thereby confined inside the polypyrrole. The glucose oxidase had a strong connection with the film of polypyrrole, allowing for the transduction of signals. An amperometric approach was used to determine the electrical current generated during the process of oxidation of hydrogen peroxide (H_2_O_2_), a byproduct of the electrochemical reaction [235].

By utilizing various conducting polymers, Abidian and colleagues reported a different kind of glucose biosensor. As a result of PEDOT’s great chemical stability and excellent conductivity, it was used in this novel glucose biosensor. PEDOT nanofiber and film biosensors were produced electrochemically on platinum (Pt) neural microelectrodes. On the surface of the Pt microelectrode arrays, a PEDOT film was directly deposited. Electrodeposition of PEDOT on Pt microelectrodes and around electrospun nanofibers resulted in the synthesis of PEDOT nanofiber biosensors. During electro-polymerization procedures, the glucose oxidase enzyme was immobilized on PEDOT films and nanofibers. Electrochemical quartz crystal microbalance (EQCM) was used to compare the differences in the incorporation of glucose oxidase between the two biosensors. When more glucose was introduced to the electrolyte solution, two biosensors were tested for sensitivity by measuring the amperometric change in the current. They discovered that biosensors made of PEDOT nanofiber exhibited higher sensitivity and more glucose oxidase integrated into the nanofibers than PEDOT film biosensors [236,237].

Boron-doped diamond (BDD) DNA biosensors were studied by Gu et al. using polyaniline/polyacrylate modifications. Using an electrochemical polymerization process, a thin coating of polyaniline/polyacrylate was applied on the surface of diamond electrodes. The DNA-sensing probes [238] were immobilized using carboxylic groups in polyaniline/polyacrylate. DNA with nucleotide sequences that were complementary to the DNA probes might be detected by DNA probes.

CNTs can also be used to alter biosensors made of conducting polymers. For instance, Luo et al. modified a PANI-based biosensor using CNTs. The PANI biosensor’s mechanical durability and conductivity may be improved by the CNTs. In this work, it was shown that CNTs might increase the effectiveness of enzyme immobilization and absorption. During electrochemical polymerization using glassy carbon electrodes, horseradish peroxidase (HRP) was incorporated into the layer of PANI. Then, by dropping dimethyl formamide with carbon nanotubes evenly dispersed at one of the electrode’s surfaces, carbon nanotubes were introduced to the surface. Researchers discovered that the addition of carbon nanotubes to the PANI electrodes boosted the amount of HRP that was already integrated into the biosensors. Upon comparing the performance of the PANI/CNTs and PANI biosensors, it was discovered that the biosensor containing carbon nanotubes had a greater signal, possibly because of the stable bonding of HRP when combined with CNTs and PANI [239,240].

### 8.3. As Bioactuators

Conductive polymers can potentially be employed as biomedical actuators due to the change in volume that happens during the process of oxidation and reduction reactions [241]. Actuators made of CPs have been used in biomedical devices [242] and artificial muscles [243]. CP actuators are excellent candidates for artificial muscles because they offer benefits, such as being easily microfabricated, lightweight, possessing excellent strength, needing lower voltage for actuation (1 V or less), being controlled electrically, being placed continuously between the minimum and maximum values, and have a larger strain, that is advantageous for volumetric and linear actuators [241]. The potential for PANI, PPy, and composites of PPy—PANI as well CNT composites such as PANI—CNT—PPy and PANI—CNT, have all been investigated. Composites of PPy-PANI achieved the most work per cycle among other materials, which is essential for strong mechanical characteristics. Additionally, polypyrrole actuators have been developed, which utilize the forces generated by undoped and doped conductive polymers to produce movements and develop artificial muscles. The strengths of CPs, their capacity to operate at physiological or ambient temperatures, and their compatibility with liquid electrolytes similar to human fluids make them ideal for bioactuator applications [244,245,246].

Bilayer-conducting polymer actuators were developed by Otero and coworkers as potential artificial muscle devices. The bilayer structure was made up of one CP (PPy) layer and one nonconducting layer of plastic. On a stainless-steel electrode (square-shaped), the Ppy was electrochemically polymerized. The bilayered polymer was then removed from the surface of the stainless-steel electrode after being adhered to the PPy layer with plastic tape. The PPy underwent oxidation and a dimensional change when an external voltage of 1 V was added. The non-conductive plastic tape’s constant volume caused the bilayer to flex. Different currents (5–25 mA) were utilized to examine the actuator’s response time at varied stimulation intensities. Later, when submerged in an electrolyte solution, researchers were able to develop a three-layered artificial muscle constructed of PPy that could push a 6000 mg object [247].

Mazzoldi and co-workers designed steerable catheters that might be utilized in minimally invasive surgery as an optical endoscope. A steerable catheter can navigate around a tight curve or around a divergence in human vasculature. The researchers used a composite of PANI fibers and an elastomeric matrix made of solid polymer electrolyte. When two distinct areas of the same end of the catheter were subjected to two opposing electrical stimulations, one side of the catheter would contract, and the other side would expand, causing the bending of the catheter [242].

### 8.4. For Tissue Engineering

Tissue engineering refers to the “application of cells, biomaterials, and relevant molecular or physical variables, alone or in combination, to repair or replace tissue to enhance the clinical outcome”. One of the best approaches to regenerative medicine is tissue engineering [248]. Even though CPs have a number of benefits over other materials due to their electrical properties, there remains potential for improvement when aiming for tissue engineering applications [75]. By combining a scaffold, cells, and biological elements, tissue engineering aims to develop biomaterials that can restore, maintain, or improve tissue function. Biodegradable conductive polymers are extensively desirable for tissue engineering applications due to their desirable characteristics, such as controlled biodegradability, high electrical conductivity, redox stability, and three-dimensional (3D) architecture.

Synthetic organic CPs, such as PPy, PTh, PANI, and PEDOT, have undergone extensive evaluation as biomaterial scaffolds for their applications in tissue engineering due to their biocompatibility and superior electrical conductivity. It has been found that a wide variety of cells, including brain cells, muscles, and bones, are highly responsive to electrical stimulation. The electrical conductivity of CPs is a potentially helpful cue that facilitates localized electrical stimulation of tissues and cells for their growth and differentiation. For instance, numerous reports have indicated that PPy enhances neuronal activity, neurite extension, and axonal outgrowth. According to the studies, Ppy was one of the first conductive polymers whose effects on mammalian cells were investigated [249]. Further, it was observed that endothelial cells [123,249], rat pheochromocytoma cells (PC12) [250], support cells, and neurons adhered to and proliferated along with mesenchymal stem cells, keratinocytes, and primary neurons in the dorsal root ganglia (DRG) [251,252,253,254]. Numerous studies have demonstrated that the biocompatible Ppy, upon doping with pTS (Ppy/pTS), can alter the response at the cellular level for electrical stimulation. The majority of these researchers depend on the passive adsorption of serum biological molecules or the deposition of pure protein solutions at the polypyrrole surface. The effectiveness of CPs in promoting cell development and regulating cell activity was investigated. Aortic endothelial cells were cultivated on fibronectin (FN)-coated polypyrrole films and exposed to oxidizing potentials [249]. Oxidized polypyrrole encourages the spreading of cells since the decrease in the concentration of polypyrrole in its neutral condition causes the rounding of cells and a corresponding fall in the synthesis of DNA (98%). On both oxidized and neutral PPy, the viability of the cell and adhesion was excellent despite the morphological alterations.

Recently, PANI is also being explored for its application in tissue engineering. Leucoemeraldine (LM), nigraniline (NA), and emeraldine (EM) films and PANI powders were implanted into male Sprague Dawley rats by Wang et al. The rats had 19–50 weeks of dorsal skin exposure to assess the in vivo tissue response. After 50 weeks, they observed that the inflammation caused by the various forms of PANI was minimal. They examined the tissues’ histology 24 weeks following the implantation. They concluded that there was no considerable inflammation at the site of the implant and that there were no abnormalities in the muscle or adipose tissues close to the implants [255]. H9c2 cardiac myoblast adhesion and proliferation characteristics on a conductive polyaniline substrate were studied by Bidez et al. PANI was discovered to be biocompatible in both its conductive and non-conductive forms, enabling cellular adhesion and proliferation. Their research demonstrated the possibility of utilizing polyaniline as an electroactive scaffold in the field of brain and cardiac tissue engineering and demonstrated the potential of PANI as an electroactive material in the growth of excitable cells [256]. While PPy and PANI continue to be the most thoroughly examined conducting polymers in the field of tissue engineering to date, the utilization of a few other conductive polymers, such as PTh, has also been investigated.

### 8.5. As Neural Probe

The development of optimal neural electrodes can benefit from the advancements made by conductive polymers in tissue engineering, particularly concerning neurons. In this area of research, it is very important to closely link neural tissue with electrodes and properly transmit signals between both the electrode and cells because it enables flawless device integration into the network of native neuronal signaling. Conductive polymers are an ideal choice for integrating neurons with electrodes due to their high surface area, which aids in promoting an effective exchange of ions between the surrounding tissue and recording sites. The objective is to enhance the surface area of the site of recording while maintaining a minimal geometric region by isolating the action potential from a single neuron. A large surface area increases capacitance, which decreases impedance and improves the signal-to-noise ratio (SNR).

A neural probe would ideally enhance the number of neural signals recorded, maintain high capacitance, decrease noise, and remain for a prolonged duration of time. Even though polypyrrole is commonly investigated as a coating material for neural probes, most recent research has focused its attention on PEDOT due to its stable oxidative condition and superior conductivity [75]. Martin et al. modified the electrodes used for neural recording in their investigation by coating them with PPy. In this study, PPy, a silk-like polymer with fibronectin (SLPF) fragments, and nano peptides CDPGYIGSR were electrochemically coated onto the gold (Au) electrode sites of a neural probe. They cultured human neuroblastoma cells and rat glial cells on the surface of neural probes with uncoated and coated electrodes. They noticed that glial cells attached more quickly to an electrode coated with PPy/SLPF than to untreated gold electrodes and that human neuroblastoma cells could grow more effectively on the electrode coated with PPy/CDPGYIGSR [257].

Abidian and co-workers coated the microelectrodes with PEDOT NTs to examine the in vivo signal recording of neural electrodes effectively. PEDOT nanotubes were synthesized through electrochemical polymerization of PEDOT on an Au site and inside and around electrospun PLLA nanofibers, which were then dissolved. They implanted the modified microelectrodes into the rat is barrel cortex. Electrochemical impedance spectroscopy was used to assess the impedance of neural electrodes at both uncoated and coated sites before and after implantation. After 7 weeks of implantation, they observed that the PEDOT nanotubes had a significantly decreased impedance than the uncoated microelectrodes denoting improved signal recording. A change in impedance was observed due to biological response and protein absorption at the neural-electrode interface. The percentage of PEDOT NT sites with quality units (Signal-to-noise ratio > 4) was 35% greater than uncoated sites over the entire course of implantation, indicating that PEDOT nanotubes recorded the activity of the brain at a higher SNR [258].

### 8.6. In Wound Healing

The provision of effective wound care is becoming increasingly important. A wound treatment methodology based on the conducting nature of human skin has arisen in the past decade and gained significant attention due to its high efficiency, ease of handling and management, and flexibility in processing [259]. Incorporating electroactive conducting polymers into the polymeric biomaterial is the fundamental step in creating conductive wound dressing [260,261]. The incorporation of CPs into wound dressings has enhanced antibacterial activity, facilitated cell proliferation, and enabled controlled drug release or electrical stimulation through the application of an external electric current [262]. So far, a lot of conductive wound dressings have been designed in various forms, such as films, membranes, hydrogel, electrospun nanofiber, etc. [262,263,264]. In addition, since tissue engineering and regenerative medicine have advanced, delicately developed conducting scaffolds resembling ECM loaded with bioactive cells or molecules have been developed for more severe wounds, such as open and chronic wounds with poor regenerating abilities, offering mechanical support and regulating cellular activities [265,266].

Shi et al. employed a w/o emulsion technique to synthesize PPy nanoparticles, which they then incorporated into PLLA [267]. The proliferation, spreading, and cell attachment of cutaneous fibroblasts of humans with or without ES was supported by this conductive composite film. Additionally, when the composite film was subjected to a direct electrical field current of 100 mV mm^−1^, cell survival was noticeably higher. Later, they investigated in detail how the PPy/PLLA layer promotes wound healing. Through the regulation of cytokines, such as IL-6 and IL-8, the conducting PPy/PLLA film under ES mediation (100 mV mm^−1^) may lengthen the survival of human cutaneous fibroblasts [268]. Mahmoud and co-workers developed a PPy/PLA membrane that was doped with heparin [269]. When exposed to ES, the conductive film demonstrated enhanced activity of dermal fibroblast with increased FGF-1 and FGF-2 expression as well as accelerated myofibroblast transdifferentiation with elevated amounts of smooth muscle actin. The ability of the PPy conductive composite film to regulate the expression of various genes related to wound healing was later genetically proven. PPy can also be used to develop electronically controlled drug delivery devices since it reacts to electrical signals [270].

## 9. Conclusions

CPs offer a variety of desirable features, including sufficient electrical conductivity, excellent stability, the ability to entrap and release biomolecules, and a variable side chain structure with a highly π-conjugated molecular backbone. They can also potentially be modified for a specific purpose to improve their physical, chemical, electrical, and biocompatibility characteristics. To overcome the barriers of CPs, such as biological compatibility, poor mechanical characteristics, and low processability, researchers are investigating different chemical modification techniques to blend the non-CPs and CPs. Upon combining the conductivities of metals and flexibilities of polymers, the conductive polymers and their composites are ideal candidates in DDS and implants as they exhibit both polymeric and other characteristics necessary for the optimal functionality of these polymers. CPs are widely employed due to their characteristic features, such as biocompatibility, and their potential to be precisely controlled, targeted, and modified for designing novel DDS. They have potential applicability in the field of biosensors, bioactuators, scaffolds in tissue engineering, neural prosthetics, etc. They can also be integrated with metallic electrodes and hydrogels to be used as a composite biomaterial for employment in drug delivery, electrical stimulations, and tissue engineering fields.

## 10. Future Prospects

Conductive polymers are ideal candidates in the field of biomedicine and DDS due to their potential applications in tissue engineering scaffolds and brain stimulation. Due to their tendency of biological decomposition, bioabsorbability represents one of the main limitations. Nevertheless, in order to advance the latter stages of development, more thorough in vivo research must be carried out. Critical toxicity assessment is also necessary to advance to clinical trials on humans. Research of conductive polymers for human use is still challenging.

The fabrication of conductive polymeric films is considered to be the cheapest and quickest process; however, most films are fragile, possessing poor mechanical stability. Further, by enhancing the surface-to-volume ratio for the encapsulation of the drug, the retention time of the drug can be improved. Sophisticated CPs have now been developed with a trigger-responsive regulated release that depends on electrochemical signals. So far, the most remarkable DDSs are devices that are self-powered and built on magnesium substrates because they do not utilize expensive, complex electronic device systems to detect changes in the environment in real-time to trigger the release.

Futuristic devices will dominate current devices in that they will be able to achieve flexible electronics, making it easier for them to be properly integrated into the human body. Furthermore, devices with enhanced sensing capabilities for stimuli such as heat, pH, mechanical stress, and neuronal recording can be advocated for estimating the quantity of drug release. The utilization of conductive polymers as nano-reservoirs will encourage the development of novel drug-triggering systems, which will have the potential of a self-regulated feedback mechanism system.

## Figures and Tables

**Table 1:** A brief overview of the doping methodology.

Type of Doping	Advantages	Controlled Parameters	Disadvantages	Ref.
Electrochemical Doping	Doping/de-doping can be reversed and clean polymer can be recovered. Many dopant species can be employed. The doping level is controlled via an electrochemical cell that has a controlled amount of current passing through it.	Amount of current passed.	Electrical conductivity deterioration could result from unexpected structural distortion.	[27]
Chemical Doping	One of the easiest methods for obtaining doping is submerging the sample in a solution containing the dopant or subjecting it to a dopant vapor.	Exposure time to dopant. Vapor pressure.	Unexpected structural distortion may cause electrical conductivity decay. Low reversibility due to de-doping or doping. Performed at a slow rate to avoid inhomogeneous doping.	[28]

**Table 4:** Brief overviews of various DDSs incorporating conductive polymer-based nanoparticles.

Drug	Type of Dopant	Polymer Type	Synthesis	Release Mechanism	Ref.
Fluorescein sodium salt and methotrexate (1–2.2 mM)	Sodium dodecyl sulfate (0.1 M)	Polypyrrole	Various types of oxidizing agents (H_2_O_2_, FeCl_3_, HAuCl_4_) were utilized to synthesize NPs made of polypyrrole. Drugs were incorporated into the reaction prior to the formation of particles.	Stimuli were applied every 3 min at a constant potential of −0.5 V for 20 s.	[198]
Fluorescein, piroxicam and insulin (3 mg/mL)	Sodium dodecyl sulfate (0.1 M)	Polypyrrole	Chemical polymerization in an aqueous medium by encapsulating the drug in the reaction mixture.	Constant electrical current was applied at −50, −100, −200, and −300 μA.	[202]
Fluorescein and daunorubicin (1–2 mM)	Dodecyltrimethylammonium bromide (0.162 M) and decanol (0.236 M)	Anionic and cationic Polypyrrole	NPs were produced via polymerization process in aqueous medium containing FeCl_3_ as oxidant, surfactant and the drugs to the reaction mixture	Fluorescein: Maintained at constant potential for 10s at −0.5 or −1.5 V and repeated at the interval of every 5 min. Daunorubicin: Maintained at constant potential for 10 s at 0.5 V, applied at the end of every 5 min.	[203]
Curcumin (0.027 M)	Sodium dodecyl sulfate (0.009 M)	Neutral PEDOT	NPs were synthesized by the process of chemical polymerization and by incorporating ammonium persulfate in the aqueous medium containing monomer, surfactant, and drug.	Constant potential was applied for 3 min at 0.50, −0.50, −1.00, and −1.25 V.	[204]
Insulin (5 mg/mL)	Sodium dodecyl sulfate (0.1 M)	Polypyrrole	PPy NPs were prepared utilizing oxidizing agent (H_2_O_2)_. The encapsulation was achieved by addition of insulin to a mixture of nanoparticles	For 20 min a constant potential of −1 V was applied.	[205]

**Table 5:** Various types of biosensors utilizing conductive polymers.

Type of Sensing Material	Type of Sensor	Polymers	Ref.
Urea (urease)	Amperometric. Conductometric. Potentiometric.	Ppy	[222]
Cholesterol (cholesterol oxidase/esterase)	Amperometric.	PPy PANI	[179,223,224]
Glucose (glucose oxidase)	Potentiometric. Amperometric	PPy PANI PTh	[225,226]
DNA (DNA hybridization)	Gravimetric Amperometric	PPy PTh	[227,228,229]
L-lactate (lactate oxidase/dehydrogenase)	Amperometric	PPy PANI PTh	[230,231]

PPy: polypyrrole; PANI: polyaniline; PTh: polythiophene.

## Data Availability

This study did not report any data.
